# Primate Infectious Disease Ecology: Insights and Future Directions at the Human-Macaque Interface

**DOI:** 10.1007/978-3-030-27920-2_13

**Published:** 2019-07-23

**Authors:** Krishna N. Balasubramaniam, Cédric Sueur, Michael A. Huffman, Andrew J. J. MacIntosh

**Affiliations:** 1 School of Resources and Environmental Engineering, Anhui University, Hefei, Anhui China; 2 Department of Biological Sciences, Primate Behavior and Ecology Program, Central Washington University, Ellensburg, WA USA; 3 Behavioral Ecology and Sociobiology Unit, German Primate Center, Leibniz Institute for Primate Research, Göttingen, Germany; 4 Department of Population Health and Reproduction, School of Veterinary Medicine, University of California at Davis, Davis, CA USA; 5 IPHC, UMR 7178, Université de Strasbourg, CNRS, Strasbourg, France; 6 Primate Research Institute, Kyoto University, Kyoto, Japan

## Abstract

Global population expansion has increased interactions and conflicts between humans and nonhuman primates over shared ecological space and resources. Such ecological overlap, along with our shared evolutionary histories, makes human-nonhuman primate interfaces hot spots for the acquisition and transmission of parasites. In this chapter, we bring to light the importance of human-macaque interfaces in particular as hot spots for infectious disease ecological and epidemiological assessments. We first outline the significance and broader objectives behind research related to the subfield of primate infectious disease ecology and epidemiology. We then reveal how members of the genus *Macaca*, being among the most socioecologically flexible and invasive of all primate taxa, live under varying degrees of overlap with humans in anthropogenic landscapes. Thus, human-macaque interfaces may favor the bidirectional exchange of parasites. We then review studies that have isolated various types of parasites at human-macaque interfaces, using information from the Global Mammal Parasite Database (GMPD: http://www.mammalparasites.org/). Finally, we elaborate on avenues through which the implementation of both novel conceptual frameworks (e.g., Coupled Systems, One Health) and quantitative network-based approaches (e.g., social and bipartite networks, agent-based modeling) may potentially address some of the critical gaps in our current knowledge of infectious disease ecology at human-primate interfaces.

## Introduction

The expansion of human populations has increased interactions and conflict between humans and nonhuman primates (hereafter primates) throughout their range. Assessing the causal factors and thereby mitigating such conflict pose a major challenge for anthropologists, primatologists, and conservation biologists. This is because human-primate interactions are spatiotemporally variable in form and frequency (reviewed in Dickman 2012; Paterson and Wallis 2005). For instance, some of these interactions include (1) human-induced changes to primate habitat that lead to the fragmentation and decline of primate populations [e.g., Zanzibar red colobus monkeys (*Procolobus kirkii*): Siex 2005], (2) increased crop-raiding by primates leading to transactional costs on humans [e.g., Buton macaques (*Macaca ochreata*): Priston et al*.* 2012], (3) human-primate competition for space and resources [e.g., chimpanzees (*Pan troglodytes*): Hockings et al*.* 2012], (4) injuries to both humans and primates on account of direct aggression [e.g., rhesus macaques (*Macaca mulatta*): Southwick and Siddiqi 1994, 1998, 2011], and (5) primate-induced damage to human property and landscapes that generate transactional or opportunity costs to humans (Barua et al*.* 2013).

In comparison to such readily discernible negative effects, one outcome of conflict that is subtler and hence often goes undetected or unchecked is the acquisition and transmission of infectious diseases (Barua et al*.* 2013; Wolfe et al*.* 2007). Our shared evolutionary histories, along with physiological and behavioral similarities, make many primate species natural reservoirs of human parasites (Fiennes 1967; Nunn and Altizer 2006; Tutin 2000). Likewise, the acquisition of parasites from humans has led to disease outbreaks among free-living primates (Kaur and Singh 2009; Kaur et al*.* 2008, 2011; Nunn and Altizer 2006). From an ecological standpoint, free-living primates may acquire parasites from humans in many ways. For example, increasing epidemiological assessments continue to establish the sharing of parasites between humans and populations of socioecologically flexible primates like baboons and macaques which have become increasingly reliant on human-provisioned food or garbage in areas of overlap (Engel and Jones-Engel 2011; Engel et al*.* 2008; Jones-Engel et al*.* 2005). Humans may also indirectly influence primate exposure to parasites by altering the environment, which may potentially subdivide primate populations and change their behavioral and foraging strategies (Chapman et al*.* 2005, 2006a; Huffman and Chapman 2009). Third, wild primates may also sometimes be exposed to “spillovers” of parasites from international travelers during ecotourism and biological field research (Carne et al*.* 2017; Engel et al*.* 2008; Jones-Engel et al*.* 2005; Marechal et al. 2011; Muehlenbein and Ancrenaz 2010). Such a wide range of potential disease acquisition and transmission routes make human-primate interfaces hot spots for emerging infectious diseases (EIDs) (Nunn et al*.* 2008; Wolfe et al*.* 2007). This is especially significant in the light of the growing call for a global, transdisciplinary strategy to deal with zoonoses in both humans and animals (the One Health, hereafter OH, concept: Destoumieux-Garzon et al*.* 2018; Zinsstag et al*.* 2011, 2015). Finally, human activities like agricultural and urban land development, tourism, and provisioning, aside from directly influencing exposure as stated above, may also influence variation in the susceptibility of primates to parasites once exposed, for example, by altering levels of stress and immune function (Chapman et al*.* 2006b; Marechal et al*.* 2011, 2016; Muehlenbein and Ancrenaz 2010).

In this chapter, we focus on how human-macaque interfaces, being hot spots for the transmission of a diverse array of parasites, present opportunities for human-primate infectious disease ecology research. We first briefly outline the significance and primary objectives behind research on primate infectious disease ecology, highlighting the greater focus to date on research implementing such approaches to study wild primates in comparison to research at human-primate interfaces. We next reveal how macaques, and more broadly the variable nature of human-macaque interfaces, present opportunities to study human-primate disease transmission from a socioecological perspective (Engel and Jones-Engel 2011; Jones-Engel et al*.* 2005; Nahallage and Huffman 2013). We then provide a detailed account of previous studies we extracted from the online Global Mammal Parasite Database (Nunn and Altizer 2005; Stephens et al*.* 2017) that have detected parasites at human-macaque interfaces. Finally, we demonstrate how the implementation of novel conceptual frameworks like the Coupled Natural and Human Systems (An and Lopez-Carr 2012; Destoumieux-Garzon et al*.* 2018; Liu et al*.* 2007) and One Health concepts (Destoumieux-Garzon et al*.* 2018; Zinsstag et al*.* 2011, 2015), as well as the implementation of cutting-edge methodological approaches like Social Network Analyses (e.g., Drewe and Perkins 2015; Pasquaretta et al*.* 2014; Rushmore et al*.* 2017; VanderWaal and Ezenwa 2016) and community-level bipartite or multimodal Networks (e.g., Dormann et al*.* 2017; Gomez et al*.* 2013; Latapy et al*.* 2008), can address some of the critical gaps in these studies to offer key future directions for epidemiological research at these interfaces.

## Primate Infectious Disease Ecology

An infectious disease is a disorder that is caused by an infectious agent, or in ecological terms a “parasite,” that causes pathology in its host (MacIntosh 2016). In the ecological realm, a “parasite” is considered any organism that lives within (or on) another “host” organism, at some cost to the latter (MacIntosh 2016). For the remainder of the chapter, we deal with enteric parasites or “endoparasites” (hereafter just “parasites”), which live within the body of the host organism. These typically fall under seven major types of organisms. Five of these, specifically bacteria, viruses, rickettsia, prions, and fungi, are conventionally pathogenic microorganisms. The last two, protozoa and helminths, include both pathogenic and non-pathogenic species. All parasites typically disrupt the normal, homeostatic functioning of the body, both directly as a result of their own activity and indirectly by stimulating the host’s immune system to produce a defensive response. They may do so by their sheer presence, by competing with host cells and symbiotic microbes, and, in extreme cases, by releasing toxins that increase the severity of diseases. Depending on their ecologies or life histories, parasites may enter hosts via their exposure to contaminated environmental sources such as food, water, and soil (e.g., enteric bacterial pathogens: Kilonzo et al*.* 2011; Sinton et al*.* 2007). They may also spread rapidly through host populations via mechanisms such as (1) direct host-to-host contact (e.g., respiratory viruses), (2) the sharing of common, contaminated environmental space or resources (e.g., enteric bacteria such as *Salmonella* sp*.*, *Shigella* sp*.*), (3) exchange of body fluids (e.g. blood-borne pathogens like HIV and HPV), or via (4) vector-borne transmission [e.g., mosquitoes spreading malarial parasites (*Plasmodium* sp*.*)] (summarized in Engel and Jones-Engel 2011; Nunn and Altizer 2006).

Infectious disease ecology is a subfield that deals with the evolutionary and environmental factors that influence the exposure, acquisition, and transmission dynamics of parasites within and (more recently) between human and animal populations (Grenfell and Dobson 1995; Hudson et al*.* 2002). As we have now entered the Anthropocene epoch, human influence on the environment has generated an increased awareness of the importance of both public health and the conservation of natural ecosystems. So it is not surprising that over the last two decades in particular we have seen an incredible surge in research related to infectious disease ecology and evolution (reviewed in Huffman and Chapman 2009; Kappeler et al*.* 2015; MacIntosh and Frias 2016; Nunn 2012; Nunn and Altizer 2006), with interdisciplinary approaches drawing on theory and methods from several biological sciences including anthropology, evolutionary genetics, behavioral ecology, epidemiology, network theory, and statistics.

Nonhuman primates have served as especially useful model host systems in these endeavors (summarized in Huffman and Chapman 2009; Nunn 2012; Nunn and Altizer 2006). In addition to sharing evolutionary histories and, increasingly, ecological space with humans, primates also exhibit diverse forms of social systems, characterized by heterogeneity in group composition and size, dispersal patterns, foraging strategies, mating systems, and social structures (Hinde 1976; Kappeler and Van Schaik 2002; Sterck 1998; Thierry 2007a). For these reasons, they are physiological, ecological, and behavioral model host systems for infectious disease research (MacIntosh 2016). There is now consensus among scientists that the evolutionary, ecological, and social diversity of free-living primates is impacted by (or indeed impact) the risk of acquisition and transmission of parasites (Sueur et al. 2018).

Broadly, empirical research on primate infectious disease ecology to date has had five major foci. First, in studies related to (1) *parasite-host co-evolution*, evolutionary anthropologists have attempted to establish links between the phylogenetic relationships of parasites and their primate hosts (MacIntosh and Frias 2016; Nunn 2011; Nunn and Altizer 2006; Petrášová et al*.* 2011; Vallo et al*.* 2012). Second, studies on (2) *primate parasite socioecology*, in addition to the relative role(s) of resource abundance, predation pressure, and infanticidal risk, have also begun to examine the role of parasites in shaping the evolution of primate group sizes and social network structure (Chapman et al*.* 2009; Nunn et al*.* 2011; meta-analyses by Griffin and Nunn 2012; Nunn et al*.* 2015; Patterson and Ruckstuhl 2013; Rifkin et al. 2012). Conversely, the idea that group-living and social structure may also impact the diversity and prevalence of parasites in hosts (Drewe and Perkins 2015; VanderWaal and Ezenwa 2016) has led to such socioecological approaches to also focus on the identification of potential “super spreaders” or “social bottlenecks” of infection (Balasubramaniam et al*.* 2016, 2018; Duboscq et al*.* 2016; Griffin and Nunn 2012; MacIntosh et al*.* 2012; Romano et al*.* 2016)*.* Other studies have used agent-based models to predict the prevalence and transmission of parasites through artificial primate groups and networks (Griffin and Nunn 2012; Nunn et al*.* 2015). Yet social life does not always equate to disease transmission or threats to homeostasis. Indeed, studies on both captive and wild primates that assess the links between (3) *infection risk and sociality, stress, and immune function* have tested the opposite paradigm, i.e., that possessing strong, diverse social connections, rather than increasing the risk of pathogenic acquisition, may function to socially buffer some primates against infection (Balasubramaniam et al*.* 2016; Duboscq et al*.* 2016; Sapolsky et al*.* 2000; Young et al*.* 2014). More research has focused on the impact of (4) *parasites in primate conservation and management*—while some deal with the implications of introduced species on the prevalence and diversity of parasites in indigenous primates (Petrášová et al*.* 2010, 2011), other research has attempted to quantify differences in parasite richness or diversity in primates living in varying degrees of human influence or in relation to their threatened status(es) (Bublitz et al*.* 2015; Chapman et al*.* 2006a; Gillespie et al*.* 2005; Goldberg et al*.* 2007; Kowalewski et al*.* 2011). Finally, emerging lines of research have focused on (5) *primate counter-strategies*, including avoidance behaviors to minimize exposure to parasites (Amoroso et al*.* 2017; Poirotte et al*.* 2017, 2019; Sarabian and MacIntosh 2015; Sarabian et al*.* 2017), and self-medication that removes or minimizes the impact of an infection on the host (Huffman 2016).

To date, much of the empirical work related to primate infectious disease ecology has focused on wild or red-listed primate populations (reviewed in Frias and MacIntosh 2018). Aside from habitat loss and fragmentation (Hussain et al*.* 2013), red-listed populations also face the risk of extinction on account of infectious diseases transmitted from humans or livestock (reviewed in Frias and MacIntosh 2018). In comparison, less research has focused on the relationship between host socioecology and transmission of parasites between humans and free-living primates at overlapping interfaces (Kaur and Singh 2009). This is despite the wide recognition that humans and primates strongly influence each other’s biology, behavior, and health (Fuentes 2012; Fuentes and Hockings 2010) and that such human-primate interfaces are also potential sources of EIDs (Jones-Engel et al*.* 2005; Nunn et al*.* 2008; Wolfe et al*.* 2007).

## Human-Macaque Interfaces

The genus *Macaca* is the most diverse, geographically widespread, and ecologically successful group of primates (Cords 2013; Thierry 2007a, b). They constitute 23 extant species, which range from North Africa in the West (Barbary macaques: *M. sylvanus*), across the Indian subcontinent [e.g., rhesus macaques (*M. mulatta*), bonnet macaques (*M. radiata*)], China [e.g., rhesus macaques (*M. mulatta*), Tibetan macaques (*M. thibetana*)], and Southeast Asia [e.g., long-tailed macaques (*M. fascicularis*), Sulawesi macaque species (e.g., *M. nigra*, *M. tonkeana*)], and up to Japan in the Far East [Japanese macaques (*M. fuscata*)] (Thierry 2007a, b). Across this range, their ecological flexibility is evidenced by the fact that macaque species, and indeed populations of the same species, inhabit a wide variety of habitats, from tropical rainforests to snowcapped mountains and from dry scrub forests to urbanized human settlements (Cords 2013; Gumert et al*.* 2011; Thierry 2007a, b).

In nature, all macaque species show broadly similar social organization [but see Sinha et al*.* (2005) for an exception]—they live in multi-male multi-female social groups in which females are philopatric and males disperse from their natal groups (Cords 2013; Thierry 2013). At the same time, they show a remarkable degree of inter- and intraspecific variation in the structure of social relationships, ranging from despotic, nepotistic societies with steep dominance hierarchies and modular, centralized, and kin-biased social networks (e.g., rhesus macaques, Japanese macaques) to tolerant or egalitarian societies characterized by shallower dominance relationships and dense, decentralized, and well-connected social networks (e.g., Sulawesi crested macaques: Balasubramaniam et al*.* 2012; Thierry et al*.* 2008; Sueur et al*.* 2011a).

**Fig. 13.1:**
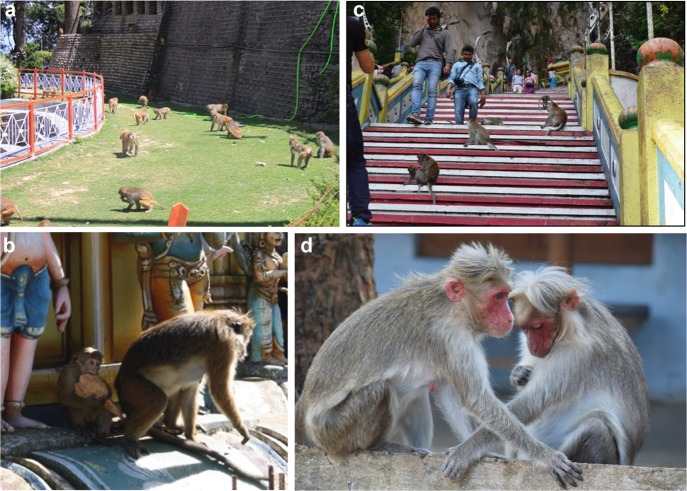
Macaques at human-macaque interfaces, specifically (**a**) rhesus macaques in Himachal Pradesh, Northern India; (**b**) long-tailed macaques in Kuala Lumpur, Malaysia; (**c**) toque macaques in Colombo, Sri Lanka; (**d**) bonnet macaques in Kerala, Southern India. Photo credits: (**a**), (**b**) and (**d**): K. N. Balasubramaniam; (**c**): M. A. Huffman

More pertinently, macaques also vary in the extent to which they show adaptive or maladaptive responses to human disturbance and anthropogenic landscapes, i.e., along a spectrum of overlap at human-macaque interfaces (Priston and McLennan 2013; Radhakrishna and Sinha 2011; Radhakrishna et al*.* 2013). At the upper end of this spectrum lie rhesus and long-tailed macaques (Fig. 13.1a, b). Large populations of these “weed” species, categorized as “Least Concern” by IUCN since 2010 (IUCN 2019), gravitate toward and even preferentially exploit human settlements (Jaman and Huffman 2013; Southwick and Siddiqi 1994, 2011; Southwick et al*.* 1983). Long-tailed macaques are even listed among the IUCN Invasive Species Specialist Group’s (ISSG) top 100 invasive species in the world (Lowe et al*.* 2000). Thus, they inhabit a variety of human-macaque interfaces: from buffer zones of ecotourism in national parks, to agricultural fields bordering rural villages, to urbanized cities like Delhi, Dhaka, and Kuala Lumpur (Fig. 13.1a, b). Other species like bonnet macaques and toque macaques (*M. sinica*) are not far behind, with both wild and semi-urban populations that inhabit the smaller town-, temple-, and university campus-interfaces of Southern India and Sri Lanka, respectively (Huffman et al*.* 2013a; Nahallage and Huffman 2013; Nahallage et al*.* 2008; Radhakrishna et al*.* 2013; Ram et al*.* 2003; Sinha et al*.* 2005) (Fig. 13.1c, d). Yet some of these species, like toque macaques, remain listed as “vulnerable” or “endangered” on account of the negative effects of ecotourism and habitat loss throughout their range (IUCN 2019). Finally, some less ecologically flexible species like lion-tailed macaques (*M. silenus*), Tibetan macaques (*M. thibetana*), and Sulawesi crested macaques (*M. nigra*) are still exposed to the negative impact of human activity in the form of habitat loss affecting their socioecology (Kumara et al*.* 2014; Singh et al*.* 2001), ecotourism-related stressors and mortality rates (Berman et al*.* 2007; Marechal et al*.* 2011), and hunting for bush-meat impacting mortality rates (Kyes et al*.* 2012; Palacios et al*.* 2012; Riley 2007; Riley and Fuentes 2011). Indeed, many of these species are classified as being “endangered” or “critically endangered” as a result (IUCN 2019).

The rise of *ethnoprimatology* as a subfield of biological anthropology has occurred simultaneously with the rise of primate infectious disease ecology. Specifically, ethnoprimatology is related to understanding how humans and primates impact each other’s niche construction, behavioral biology, and health-related outcomes (Dore et al*.* 2017; Fuentes 2012; Fuentes and Hockings 2010). Unsurprisingly, human-macaque interfaces in North Africa, India, Sri Lanka, and Southeast Asia have been the primary foci of most ethnoprimatological research, with some more recent studies in Africa also having been conducted on baboons (Fehlmann et al*.* 2016; Kaplan et al*.* 2011; Hoffman and O’Riain 2012), chimpanzees (Hockings et al*.* 2012), and lemurs (Loudon et al*.* 2017). To date, this work has revealed that the nature, frequency, and severity of interactions and conflict at human-macaque interfaces vary broadly by context (Radhakrishna and Sinha 2011; Radhakrishna et al*.* 2013). For instance, across the Indian subcontinent, China, and Southeast Asia, conflict is heavily influenced by whether macaques also play more positive roles with resident and/or visiting human communities, e.g., monkeys as religious symbols, pets, trade commodities, or tourist attractions (Jones-Engel et al*.* 2004; Nahallage and Huffman 2013; Radhakrishna et al*.* 2013). At the same time, some intrinsic characteristics of macaques, including the age-sex class‚ personalities of individuals, and/or species-typical adaptive responses, have also been shown to influence interface interactions (Beisner et al*.* 2014; Fuentes 2006; Marechal et al. 2011; Sha et al*.* 2009). Such variation in human- and macaque-specific features across interfaces generates a broad variety of direct and indirect interactions, such as (1) human provisioning of macaques, (2) macaques using anthropogenic landscape features (e.g., buildings, fences, water tanks), (3) mutual contact- and non-contact aggression, (4) the exchange of body fluids like blood and saliva, (5) the fragmentation of macaque populations on account of the loss of natural habitat, (6) the hunting and consumption of macaques by humans as bush-meat, and (7) the use of macaques as pets, trade commodities, or tourist attractions (Fuentes et al*.* 2011; Gumert et al*.* 2011; Hussain et al*.* 2013; Jones-Engel et al*.* 2004; Radhakrishna et al*.* 2013; Riley and Fuentes 2011; Riley 2003). Naturally, the dynamic nature of such environments provides myriad mechanisms for the acquisition and transmission of parasites (Engel and Jones-Engel 2011; Nunn 2012).

## Parasites at Human-Macaque Interfaces

To extract and review previous studies that report parasites among free-living macaque populations at human-macaque interfaces, we relied on the Global Mammal Parasite Database (or GMPD, Version 2.0: Stephens et al*.* 2017). The GMPD is a compilation of studies that report disease-causing organisms—bacteria, viruses, protozoa, helminths, and fungi—isolated from wild or free-living populations of some of the major mammalian taxa, specifically ungulates, carnivores, and primates (Nunn and Altizer 2005; Stephens et al*.* 2017). The database now contains 24,000 records, from over 2700 literature sources including journal articles, books and book chapters, and reports at conference proceedings. Records may be filtered on the basis of different parasite or host-specific characteristics, such as taxonomic categories, geographic location, and mode of transmission.

**Fig. 13.2:**
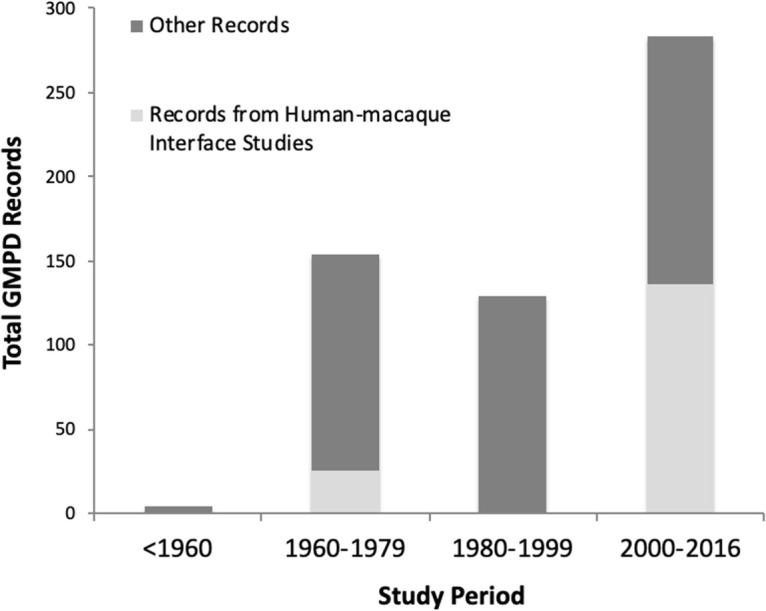
Records of parasites reported among free-living macaque populations from studies in the Global Mammal Parasite Database (GMPD)

A search of the GMPD database filtered by host genus (macaques) and type of parasite (bacteria, viruses, helminths, and protozoa) revealed 570 records from across 80 different studies. Figure 13.2 indicates the distribution of these records by study period. Aside from the general geographic location, the GMPD does not offer more specific filtering options that aid in the classification of studies in accordance with socioecological conditions under which they were conducted. So, we manually screened for “human-macaque interface” studies as those among the above studies that reported one or more of the following: (1) the direct transmission of these agents between humans and macaques in either direction (e.g., contact aggression or provisioning, pet macaques released into free-living populations), (2) the possible or potential transmission of such agents, or (3) the indirect impact of humans or anthropogenic factors (e.g., habitat fragmentation, livestock, macaque foraging on provisioned food) being identified to have influenced the acquisition of these parasites among macaques. Since this chapter primarily deals with the socioecological impact of human-macaque interfaces on disease risk, we also did not include studies on the phylogenetic co-evolutionary roots of parasites and their primate hosts.

These criteria led to the extraction of 161 (out of 570) macaque records, the vast majority of which were dated post 2000 (130 out of 161: Fig. 13.2). These records were spread across nine cited studies conducted on six different species of macaques. In Table 13.1, we summarize information from these studies, providing details on the parasites isolated, host macaque species, geographic location, prevalence and the number of individuals sampled (where the information is available), and the type of acquisition reported or speculated. Unsurprisingly, viral agents dominate this list with 88 of the 161 entries (or 55%) from 4 citations. During the last two decades, several zoonotic viruses have been described and studied in nonhuman primates in Africa and Asia from evolutionary and virulence perspectives (e.g., Ebola in great apes, reviewed in Leendertz et al*.* 2017; respiratory viruses in macaques, reviewed below). From a socioecological standpoint, studies on macaques have revealed strong associations between the frequency of intense human-macaque contact behaviors that involve the exchange of body fluids (e.g., aggressive bites and scratches) and the prevalence of respiratory viruses. Early work showed that wild-caught rhesus macaques in Northern India that had the highest degrees of exposure to human contact were also the most likely to show blood serum antibodies against human respiratory viruses (Shah and Southwick 1965). Later work in Nepal revealed correlations between the frequency of intense contact interactions with humans like aggressive bites and scratches and the seroprevalence of respiratory viruses such as the simian foamy virus (SFV), simian type-D virus, Cercopithecine herpesvirus-1 (CHV-1), and simian virus-40 (SV-40) (Jones-Engel et al*.* 2006) among rhesus macaques living in human settlements. In comparison, the Barbary macaques of Gibraltar, which engage in less intense aggression and have lower rates of contact bites in comparison to the Asian rhesus populations, showed a markedly lower seroprevalence (or were even seronegative) of these same respiratory viruses (Engel et al*.* 2008). More recently, humans traveling with performing “pet” rhesus macaques were found to indirectly influence the genetic structure and translocation of macaque SFV across rhesus populations in Bangladesh (Feeroz et al*.* 2013). Finally, other work not included in the GMPD has recorded the prevalence of macaque-borne viral pathogens like SFV and retroviruses among humans that regularly come into contact with these populations (Jones-Engel et al*.* 2005).

**Table 13.1:** Summary of studies extracted from the GMPD that have reported parasites at human-macaque interfaces

Citations	GMPD entries	Parasite	Parasite genus	Host species	Location	Lat.	Long.	Prev.	Type of acquisition (reported or speculated)
Huffman et al. (2013a)	7990	Bacteria	*Escherichia coli*	*Macaca sinica*	Sri Lanka	7	81	0.25	Direct: human-macaque interactions
Huffman et al. (2013a)	7992	Helminth	*Trichuris* sp.	*Macaca sinica*	Sri Lanka	7	81	0.22	Indirect: habitat fragmentation
Huffman et al. (2013a)	7991	Protozoa	*Entamoeba histolytica*	*Macaca sinica*	Sri Lanka	7	81	0.07	Direct: human-macaque interactions
Hussain et al. (2013)	7873	Helminth	*Ancylostoma* sp.	*Macaca silenus*	Southern India	10.4	77.02	0.26	Indirect: habitat fragmentation
Hussain et al. (2013)	7880	Helminth	*Diphyllobothrium* sp.	*Macaca silenus*	Southern India	10.4	77.02	0.02	Indirect: habitat fragmentation
Hussain et al. (2013)	7875	Helminth	*Strongyloides* sp.	*Macaca silenus*	Southern India	10.4	77.02	0.32	Indirect: habitat fragmentation
Hussain et al. (2013)	7877	Helminth	*Trichuris* sp.	*Macaca silenus*	Southern India	10.4	77.02	0.32	Indirect: habitat fragmentation
Hussain et al. (2013)	7881	Protozoa	*Balantidium* sp.	*Macaca silenus*	Southern India	10.4	77.02	0.06	Indirect: habitat fragmentation
Ekanayake et al. (2006)	5945–5948; 6090	Helminth	*Enterobius* sp.	*Macaca sinica*	Sri Lanka	7.9	81	0.51	Indirect: soil/livestock
Ekanayake et al. (2006)	5961–5964; 6094	Helminth	*Balantidium coli*	*Macaca sinica*	Sri Lanka	7.9	81	0.26	Indirect: soil/livestock
Ekanayake et al. (2006)	5969–5972; 6096	Protozoa	*Chilomastix* sp.	*Macaca sinica*	Sri Lanka	7.9	81	0.12	Indirect: soil/livestock
Ekanayake et al. (2006)	5937–5940	Protozoa	*Cryptosporidium* sp.	*Macaca sinica*	Sri Lanka	7.9	81	0.29	Indirect: soil/livestock
Ekanayake et al. (2006)	5965–5968; 6095	Protozoa	*Entamoeba coli*	*Macaca sinica*	Sri Lanka	7.9	81	0.30	Indirect: soil/livestock
Ekanayake et al. (2006)	5973–5976, 6097	Protozoa	*Entamoeba hartmanni*	*Macaca sinica*	Sri Lanka	7.9	81	0.27	Indirect: soil/livestock
Ekanayake et al. (2006)	5977–5980; 6098	Protozoa	*Entamoeba histolytica*	*Macaca sinica*	Sri Lanka	7.9	81	0.30	Indirect: soil/livestock
Ekanayake et al. (2006)	5957–5960; 6963	Protozoa	*Iodamoeba* sp.	*Macaca sinica*	Sri Lanka	7.9	81	0.27	Indirect: soil/livestock
Ekanayake et al. (2006)	5949–5952; 6091	Helminth	*Strongyloides* sp.	*Macaca sinica*	Sri Lanka	7.9	81	0.29	Indirect: soil/livestock
Ekanayake et al. (2006)	5953–5956; 6092	Helminth	*Trichuris* sp.	*Macaca sinica*	Sri Lanka	7.9	81	0.09	Indirect: soil/livestock
Engel et al. (2008)	7131; 7302; 7308; 7314; 7321	Virus	*Betaretrovirus Mason-Pfizer monkey virus*	*Macaca sylvanus*	Gibraltar	36	−5.6	0.00	Direct: human-macaque interactions
Engel et al. (2008)	7128; 7304; 7310; 7315; 7320	Virus	*Deltaretrovirus STLV 1*	*Macaca sylvanus*	Gibraltar	36	−5.6	0.00	Direct: human-macaque interactions
Engel et al. (2008)	7130; 7301; 7307; 7313; 7319	Virus	*Lentivirus SIV* sp.	*Macaca sylvanus*	Gibraltar	36	−5.6	0.00	Direct: human-macaque interactions
Engel et al. (2008)	7127; 7303; 7309; 7316; 7323	Virus	*Simplexvirus Herpes simplex virus 1*	*Macaca sylvanus*	Gibraltar	36	−5.6	0.00	Direct: human-macaque interactions
Engel et al. (2008)	7132; 7300; 7306; 7312; 7318	Virus	*Spumavirus Simian foamy virus*	*Macaca sylvanus*	Gibraltar	36	−5.6	0.59	Direct: human-macaque interactions
Feeroz et al. (2013)	7833–7845; 8102–8107	Virus	*Spumavirus Simian foamy virus*	*Macaca mulatta*	Bangladesh			0.92	Direct: human-macaque interactions, geographic isolation
Jones-Engel et al. (2006)	3729; 5422; 5458; 5521; 5581	Virus	*Cytomegalovirus Cercopithecine herpesvirus 8*	*Macaca mulatta*	Nepal	27.7	85.3	0.95	Indirect: habitat fragmentation, human provisioning
Jones-Engel et al. (2006)	3730; 5423; 5459; 5522; 5582	Virus	*Polyomavirus SV-40*	*Macaca mulatta*	Nepal	27.7	85.3	0.90	Direct: human-macaque interactions
Jones-Engel et al. (2006)	3731;5424; 5560; 5523; 5583	Virus	*Simplexvirus Cercopithecine herpesvirus 1*	*Macaca mulatta*	Nepal	27.7	85.3	0.64	Direct: human-macaque interactions
Jones-Engel et al. (2006)	3732; 5425; 5461; 5524; 5584	Virus	*Spumavirus Simian foamy virus*	*Macaca mulatta*	Nepal	27.7	85.3	0.97	Direct: human-macaque interactions
Lee et al. (2011)	7157; 7164	Protozoa	*Plasmodium coatneyi*	*Macaca fascicularis*	Borneo			0.45	Indirect: anopheline vector
Lee et al. (2011)	7160; 7161	Protozoa	*Plasmodium cynomolgi*	*Macaca fascicularis*	Borneo			0.48	Indirect: anopheline vector
Lee et al. (2011)	7159; 7162	Protozoa	*Plasmodium fieldi*	*Macaca fascicularis*	Borneo			0.02	Indirect: anopheline vector
Lee et al. (2011)	7158; 7163	Protozoa	*Plasmodium inui*	*Macaca fascicularis*	Borneo			0.80	Indirect: anopheline vector
Lee et al. (2011)	7156; 7165	Protozoa	*Plasmodium knowlesi*	*Macaca fascicularis*	Borneo			0.89	Indirect: anopheline vector
Shah and Southwick (1965)	3135; 5519; 5579	Virus	*Alphavirus Chikungunya*	*Macaca mulatta*	Northern India	28	80	0.00	Direct: human-macaque interactions
Shah and Southwick (1965)	5508; 5568	Virus	*Dengue virus group Dengue 1*	*Macaca mulatta*	Northern India	28	80	0.00	Direct: human-macaque interactions
Shah and Southwick (1965)	5516; 5576	Virus	*Enterovirus poliovirus 1*	*Macaca mulatta*	Northern India	28	80	0.00	Direct: human-macaque interactions
Shah and Southwick (1965)	5517; 5577	Virus	*Enterovirus poliovirus 2*	*Macaca mulatta*	Northern India	28	80	0.13	Direct: human-macaque interactions
Shah and Southwick (1965)	5518; 5578	Virus	*Enterovirus poliovirus 3*	*Macaca mulatta*	Northern India	28	80	0.00	Direct: human-macaque interactions
Shah and Southwick (1965)	5507; 5567	Virus	*Flavivirus Japanese encephalitis*	*Macaca mulatta*	Northern India	28	80	0.00	Direct: human-macaque interactions
Shah and Southwick (1965)	5514; 5574	Virus	*Pneumovirus Human respiratory syncytial virus*	*Macaca mulatta*	Northern India	28	80	0.00	Direct: human-macaque interactions
Shah and Southwick (1965)	5510; 5570	Virus	*Polyomavirus SV-40*	*Macaca mulatta*	Northern India	28	80	0.60	Direct: human-macaque interactions
Shah and Southwick (1965)	5511; 5571	Virus	*Respirovirus Human parainfluenza virus 1*	*Macaca mulatta*	Northern India	28	80	0.05	Direct: human-macaque interactions
Shah and Southwick (1965)	5513; 5573	Virus	*Respirovirus Human parainfluenza virus 3*	*Macaca mulatta*	Northern India	28	80	0.51	Direct: human-macaque interactions
Shah and Southwick (1965)	5512; 5572	Virus	*Rubulavirus Human parainfluenza virus 2*	*Macaca mulatta*	Northern India	28	80	0.01	Direct: human-macaque interactions
Shah and Southwick (1965)	5509; 5569	Virus	*Simplexvirus Herpes simplex virus 1*	*Macaca mulatta*	Northern India	28	80	0.44	Direct: human-macaque interactions
Wenz-Mücke et al*.* (2013)	7862; 7866	Helminth	*Oesophagostomum* sp.	*Macaca fascicularis*	Thailand	16.2	103.1	0.07	Indirect: habitat fragmentation, human provisioning
Wenz-Mücke et al*.* (2013)	7858; 7863	Helminth	*Strongyloides fuelleborni*	*Macaca fascicularis*	Thailand	16.2	103.1	0.41	Indirect: habitat fragmentation, human provisioning
Wenz-Mücke et al*.* (2013)	7859; 7864	Helminth	*Trichuris* sp.	*Macaca fascicularis*	Thailand	16.2	103.1	0.54	Indirect: habitat fragmentation, human provisioning

Gastrointestinal protozoa (36 entries out of 161, or 22%) and helminths (26 entries out of 161, or 16%) were the next most commonly reported parasites among the human-macaque interface studies examined. In nature, these are among the most commonly occurring parasites in wild primates (Huffman and Chapman 2009; Nunn and Altizer 2006). Yet, a few ecological assessments of human-perturbed landscapes have revealed that anthropogenic factors may indirectly influence their acquisition among macaques [but see Lane et al*.* (2011) who report a decrease in such acquisition]. In Sri Lanka, for instance, the prevalence of both gastrointestinal protozoan parasites like *Cryptosporidium* sp., *Entamoeba* sp., and *Balantidium coli* and nematodes like *Enterobius* sp. and *Strongyloides* sp*.* was more common among toque macaques in more human-disturbed than pristine environments (Ekanayake et al*.* 2006). Further, Huffman et al*.* (2013a) speculate that increased human impact may in part be responsible for why the prevalence of helminth parasites was lower among toque macaques sampled at lower altitudes. Increased contact with anthropogenic landscapes and human-provisioned food was strongly linked to the prevalence of *Strongyloides fuelleborni* in wild long-tailed macaques in Thailand (Wenz-Mücke et al*.* 2013). Among populations of critically endangered lion-tailed macaques in Southern India, the anthropogenic fragmentation of their natural habitat was positively correlated to the diversity of gastrointestinal helminths and protozoan parasites (Hussain et al*.* 2013).

We extracted 10 records (6% of 161 records) of the protozoan parasite *Plasmodium* sp., the causative agent of malaria, all from a single study that surveyed wild populations of long-tailed and pig-tailed macaques (*Macaca nemestrina*) in Borneo (Lee et al*.* 2011). This study revealed especially high prevalence of three *Plasmodium* species—*P. knowlesi*, *P. cynomolgi*, and *P. inui*—among the macaque populations. They also revealed that *P. knowlesi*, previously hypothesized as having been transmitted to humans via anopheline vectors from overlapping macaque populations, was derived from an ancestral malarial parasite that existed before humans came to Southeast Asia. Since then, high prevalence of *P. knowlesi* has been detected among free-living macaques at vegetation mosaics and forest fragments in other parts of Southeast Asia, including Indonesia, Cambodia, Laos, and Vietnam (Huffman et al*.* 2013b; Zhang et al*.* 2016). Malaria is now widely recognized as being a threat at human-primate interfaces (Singh et al*.* 2004). In addition to thriving macaque populations acting as natural reservoirs for these parasites, the fragmented mosaic landscapes of Southeast Asia are also highly conducive to the proliferation of mosquito vector complexes like *Anopheles dirus* and *A. leucosphyrus*, which may transmit malaria into otherwise infection-naive macaque and human populations (Moyes et al*.* 2014).

The least reported type of parasite was bacterial, with only one record speculating that anthropogenic factors may be responsible for the prevalence of *Escherichia coli* in toque macaques (Huffman et al*.* 2013a). This was more broadly reflective of the general lack of studies that have focused on the detection of bacterial pathogens in free-living primates (Kaur and Singh 2009; Nunn and Altizer 2006). Bacterial pathogens like *Salmonella* sp., *Shigella* sp., and *E. coli* O157:H7 routinely cause acute diarrheal infection among humans and domestic livestock (Gorski et al*.* 2011; Rwego et al*.* 2008; Sinton et al*.* 2007; Suleyman et al*.* 2016). They have been previously isolated from wild primate populations in Africa that live in human-perturbed, fragmented habitats (chimpanzees: McLennan et al*.* 2017; lemurs of Madagascar: Bublitz et al*.* 2015). Since they strongly overlap and rely heavily on anthropogenic resources, urban and semi-urban macaques may be natural reservoirs of these agents, with the potential to disseminate them into overlapping human and critically endangered wildlife populations. A preliminary study at human-rhesus macaque interfaces in Northern India revealed that anthropogenic factors, such as rates of human-macaque aggression and provisioning, were positively correlated with the prevalence of enteric bacteria like *Salmonella* sp. and *E. coli* O157:H7 (Beisner et al*.* 2016). This finding should lead to future assessments of the relative prevalence of enteric bacteria among humans, livestock, and other overlapping macaque populations.

Our GMPD search yielded no studies on parasites in Tibetan macaques, which is somewhat surprising. There is scope for future work to focus on parasite transmission at human-Tibetan macaque interfaces. At both Mt. Emei and Mt. Huangshan, China, where they have been best studied (Zhao 1996; Li 1999), wild Tibetan macaque groups are indeed exposed to anthropogenic factors, particularly tourism. At Mt. Emei, a Buddhist community that is visited by tourists for its temples, there is no regulation of tourist-macaque interactions. Tourists regularly hand-provision the macaques, and there have been reports of tourists suffering fatal injuries from macaque attacks (Zhao 2005). Such intense and frequent contact presents scope for the transmission of parasites. On the other hand, a primate tourism program that is currently in place at Mt. Huangshan restricts the scope for macaque-tourist interactions. This program was laid down following a period between 1994 and 2004, when a group of Tibetan macaques at Mt. Huangshan was “managed” for tourist activity by restricting their home range (Berman et al*.* 2007). Studies on this group have revealed that intragroup aggression, attacks on infants, and infant mortality rates were all much higher during periods when the group’s home range was restricted for tourist viewing than in periods prior to such activity (Berman et al*.* 2007). Later work in the mid-2000s that was conducted following the period of severe range restriction revealed that the macaques showed increased self-directed behaviors (e.g., self-scratching, yawning, body shake) as well as stress-coping social behaviors (e.g., allogrooming, body contact) when they were closer to tourists, in comparison to when there were no tourists present (reviewed in Matheson et al*.* 2013). Such tourist activity, now more controlled, may have presented or may continue to present a stressful environment to Tibetan macaques that may heighten the acquisition and transmission of parasites.

## The Future of Human-Macaque Disease Ecology

Our review of studies on human-macaque interfaces reveals the detection and confirmation of a range of parasites. Yet many of these studies, based on either symptomatic or mortality-based evidence of pathogenic infection in either humans or macaques, have inferred that disease transmission has occurred without ever having established that transmission did occur (VanderWaal and Ezenwa 2016; VanderWaal et al*.* 2014). In other words, little or no work has assessed the precise mechanisms and pathways of parasite transmission at human-macaque interfaces. In this section, we illustrate how implementing (A) the conceptual frameworks of Coupled Natural and Human Systems and One Health, in combination with (B) cutting-edge network analytical techniques, may significantly enhance our current knowledge of infectious disease transmission at human-macaque interfaces.

### (A) Unifying Conceptual Frameworks: Coupled Systems and One Health

Conflict at human-macaque interfaces may be spatiotemporally variable in form and frequency and may affect parasite transmission in dynamic and sometimes unpredictable ways. In this light, one of the biggest challenges facing research on infectious disease ecology at these, and indeed all human-wildlife interfaces, is the lack of a consensual theoretical or conceptual framework applicable across multiple types of systems.

One framework that may prove useful in this regard stems from the broader conceptualization that human interactions with nature and the environment may be viewed as dynamic, coupled systems (Liu et al*.* 2007). Since its proposition, the Coupled Natural and Human Systems (or CNHS) approach has presented a significant advancement in our understanding of human impact on abiotic and (more recently) biotic factors. Traditionally, studies examining the interactions between humans and natural phenomena have been largely reductionist in nature (summarized in Liu et al*.* 2007). They have adopted principles from biology, anthropology, geography, and environmental sciences, with an almost exclusive focus on how a single component of the human system may influence a given property of a natural system or vice versa. Further, they have tended to focus on short-term effects rather than conduct long-term assessments of the feedback effects of such interactions on both human and natural systems. Expanding significantly on these assessments, the CNHS approach explicitly acknowledges that aspects of human systems and natural systems are coupled or interlinked and must therefore be assessed as a collective whole. Multiple, dynamic components of human and natural systems are expected to influence the nature and types of interactions at interfaces, with such impact being expected to reciprocally impact long-term indicators of the overall stability, sustainability, and health of both human populations and natural components.

In its short history, studies implementing CNHS frameworks have primarily focused on the impact of humans on *inanimate, abiotic* factors (e.g., landscape ecology, climatic conditions: (Foley et al*.* 2005; Postel et al*.* 1996) and their long-term effects (e.g., via environmental degradation, natural disasters: Dilley et al*.* 2005) on human population dynamics and ecology (Liu et al*.* 2007)). In comparison, fewer studies have tackled the relationship between humans and *animate* natural systems like wildlife populations (Dickman 2010, 2012). The well-documented nature of interactions and conflict at human-macaque interfaces (reviewed above) offer opportunities to address this gap. Or conversely, the CNHS framework maybe useful to assess the mechanistic processes through which the variant nature of human-macaque interfaces may favor or inhibit the transmission of parasites across human and macaque systems.

As we allude to earlier, macaques may acquire parasites in many ways, including direct physical contact with humans or during social interactions with infected conspecifics, changes in foraging strategies induced by anthropogenic landscapes, or human-induced stressors increasing macaques’ susceptibility to infection. Implementing the CNHS approach would entail examining the relative likelihood(s) of these mechanisms and indeed whether specific (suites of) attributes of the human system (e.g., community type, history of interactions with macaques, visitors versus tourists) or the macaque system (e.g., age-sex class, group size, species-typical social style) are linked with the degree to which one type of interface interaction may be expected to prevail over another in influencing parasite acquisition. More tellingly, we reckon that the CNHS framework would finally take research on human-macaque infectious disease research beyond mere descriptions of parasites at interfaces. Expanding on these findings, a CNHS approach would naturally lead to more long-term assessments of the impact of parasite diversity and distribution on indicators of macaque and human population health (e.g., symptomatic evidence for disease outbreaks, stress-induced illness), reproductive success (e.g., the number and fitness of offspring), and survival (e.g., population demographics and infant mortality rates).

Closely related to the CNHS framework is the One Health (OH) concept (derived from the “One Medicine” concept: Schwabe 1984), or the idea that addressing the challenges surrounding human health issues cannot be dissociated from environmental health (or EcoHealth) or from veterinary medical practices associated with treating wild and domestic animals (Destoumieux-Garzon et al*.* 2018; Zinsstag et al*.* 2011, 2015). The OH concept stems from the acknowledgment that the impact of human population expansion on the environment generates negative health outcomes, such as the occurrence of chronic, non-infectious diseases in humans, human and animal exposure to environmental toxins and emerging pollutants like plastics (Kannan et al*.* 2010; Waters et al*.* 2016), as well as the emergence of infectious diseases at human-wildlife interfaces (Gomez et al*.* 2013; Hudson et al*.* 2002; Nunn et al*.* 2008; Wolfe et al*.* 2007). So, it constitutes a global strategy highlighting the need for a holistic, transdisciplinary approach in dealing with the health of humans, animals, and ecosystems (the One Health Initiative).

Since its proposition more than a decade ago, proponents of OH approaches have (with varying degrees of success) proposed to deal with some of the barriers facing infectious disease research (summarized in Destoumieux-Garzon et al*.* 2018). We highlight three as being particularly relevant to human-macaque interfaces. The first is a resolution of the extent to which the factors that influence human health outcomes overlap with those that influence the health of natural ecosystems. Human-macaque interfaces are useful to conduct such assessments. This is because many (although not all) social and environmental factors that may potentially drive parasite transmission from humans to macaques, such as direct physical contact, contaminated food or water sources, and the exchange of body fluids, are also likely to transmit agents from macaques to humans (Engel and Jones-Engel 2011; Engel et al*.* 2008; Jones-Engel et al*.* 2005; Kaur and Singh 2009).

A second barrier is related to the promotion of interdisciplinary projects that combine veterinary medical assessments to detect and diagnose infectious diseases, with ecological and evolutionary approaches to understand the relationships between parasites and their hosts (MacIntosh and Frias 2016; Nesse et al*.* 2010; Nunn and Altizer 2006). Among all the primates, macaques (particularly rhesus macaques and long-tailed macaques) continue to be the most common genus used in captivity as models for biomedical research (Hannibal et al*.* 2017; Phillips et al*.* 2014). Further, as we review above, infectious disease research among free-living macaque populations have had variant foci, ranging from the detection and diagnosis of parasites (Engel and Jones-Engel 2011; Engel et al*.* 2008; Jones-Engel et al*.* 2004, 2005), through establishing co-evolutionary links between parasites and macaque hosts (Huffman et al*.* 2013b), to assessing the social and environmental underpinnings of parasite prevalence and transmission (Duboscq et al*.* 2016; MacIntosh et al*.* 2010, 2012; Romano et al*.* 2016). The OH concept, along with our above-stated argument that human-macaque interfaces are functionally interdependent, coupled systems, would provide a means to bring such diverse foci under a single, unifying framework (Destoumieux-Garzon et al*.* 2018).

Finally, a third direction involves placing a strong emphasis on complementing strong medical and theoretical knowledge, with current advancements in methodological and data analytical approaches. Below we elaborate on how one set of approaches—Network Analyses—may be especially significant for future research on infectious disease ecology at human-macaque interfaces.

### (B) Network-Based Analytical Approaches

**Fig. 13.3:**
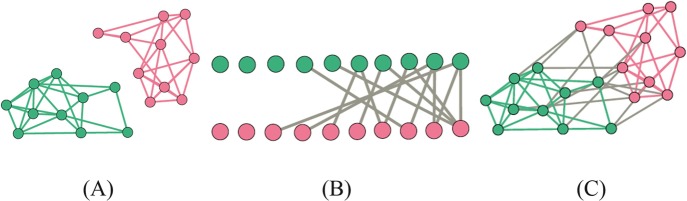
Hypothetical social (**a**), bipartite (**b**), and multimodal (**c**) networks of the same individuals. Green nodes might represent macaques and pink ones might be humans. Green links are interactions between macaques, pink links are interactions between humans, and gray links are interactions between macaques and humans (interspecies)

In the last two or three decades, network-based analytical techniques have revolutionized infectious disease epidemiology and ecology (Craft 2015; Craft and Caillaud 2011; Drewe and Perkins 2015; Godfrey 2013; Keeling 2005; Moore and Newman 2000; Newman 2002; VanderWaal and Ezenwa 2016). From a biological perspective, networks are reconstructions of entities (nodes) that are connected to each other based on one or more shared characteristics (edges) (Fig. 13.1a–c). For instance, *animal social and spatial networks* capture relationships between individuals in a social group linked together based on the frequency with which they interact or the degree of spatial overlap, respectively (reviewed in Brent et al*.* 2011; Croft et al*.* 2008; Farine and Whitehead 2015; Kasper and Voelkl 2009; Krause et al*.* 2007; Lusseau and Newman, 2004; Newman 2004; Sueur et al*.* 2011b; Wey et al*.* 2008) (Fig. 13.3a). *Bipartite or multimodal networks* add a level of complexity by distinguishing two or more components or layers of organization within a system, such that distinctions can be made between the edges that link nodes within the same layer to nodes across layers (Dormann et al*.* 2017; Kane and Alavi 2008; Latapy et al*.* 2008) (Fig. 13.3b, c). Third, links based on the degree of genotypic similarity of gastrointestinal microbes isolated from potentially interacting hosts within the same time frame may be used to reconstruct *microbial sharing or transmission networks* (VanderWaal and Ezenwa 2016; VanderWaal et al*.* 2014). Finally, in the absence or sparsity of real data, mathematical *agent-based models and artificial networks* have proven exceptionally useful in modeling the transmission of parasites (Bente et al*.* 2009; Griffin and Nunn 2012; Nunn 2009; Romano et al*.* 2016). Such heterogeneity in connectedness within and between the components of socioecological systems may strongly influence the likelihood of parasite acquisition and transmission.

Not surprisingly, network approaches, particularly social network analysis, have already found a wide range of applications in infectious disease epidemiology (Craft 2015; Craft and Caillaud 2011; Drewe and Perkins 2015; Godfrey 2013; Keeling 2005; VanderWaal and Ezenwa 2016). We briefly review these applications and related studies below. Recognizing the relative dearth in the implementation of network approaches at human-macaque interfaces, we also highlight some context(s) in which they may be implemented in human-macaque infectious disease ecological research.

### Social Networks and Parasite Acquisition

In humans and other animals, heterogeneity in space use overlap or contact social behavior may strongly influence the acquisition of parasites (reviewed in Drewe and Perkins 2015; Kappeler et al*.* 2015; Silk et al*.* 2017). Such heterogeneity can be modeled using social network analysis (Brent et al*.* 2011; Croft et al*.* 2008; Farine and Whitehead 2015; Krause et al*.* 2007; Sueur et al*.* 2011b). The first applications of social network approaches in the context of disease transmission were focused on humans, particularly in the spread of sexually transmitted diseases (or STDs) (Klovdahl 1985) and later following the detection of the severe acute respiratory syndrome (SARS) outbreak in 2003 (Meyers 2007). Since then, social networks across a wide range of taxa have been used to identify central or well-connected individuals, which may be potential “super spreaders” of parasites [e.g., lizards (*Egernia stokesii*): Godfrey et al*.* 2009; spider monkeys (*Ateles geoffroyi*): Rimbach et al*.* 2015; meerkats (*Suricata suricatta*): Drewe 2010; Japanese macaques: MacIntosh et al*.* 2012; reviewed in Drewe and Perkins 2015; VanderWaal and Ezenwa 2016]. In wild Japanese macaques, for instance, high-ranking individuals with more direct and indirect connections, or *eigenvector centrality* (Newman 2006) in their social grooming networks, were also shown to have greater species richness and infection intensities of nematode parasites (MacIntosh et al*.* 2012). Yet having strong and diverse social connections, rather than increasing parasite acquisition owing to contact-mediated transmission, may actually decrease the likelihood of such acquisition by mitigating stressors or enhancing immune function (e.g., Balasubramaniam et al*.* 2016; Cohen et al*.* 2015; Hennessy et al*.* 2009). Consistent with this “social buffering hypothesis,” work on captive rhesus macaques revealed that individuals with the strongest and most diverse social grooming and huddling connections were also the least prone to the acquisition of environmental bacterial pathogens (Balasubramaniam et al*.* 2016). Finally, aside from inter-individual differences in contact patterns, the higher order structure of social networks may also influence parasite transmission in contrasting ways. For instance, increased community modularity or substructuring in social networks, on account of individuals interacting more with subsets of preferred partners (Fushing et al*.* 2013; Newman 2006; Whitehead and Dufault 1999), may enhance parasite transmission within subgroups while presenting “social bottlenecks” to the group-wide spread of parasites (Griffin and Nunn 2012; Huang and Li 2007; Nunn et al*.* 2011, 2015; Romano et al*.* 2018; Salathe and Jones 2010). On the other hand, dense, well-connected networks, with a higher efficiency (the inverse of the number of shortest paths in the network), may facilitate rather than hinder the rapid transmission of parasites (Drewe and Perkins 2015; Griffin and Nunn 2012; Pasquaretta et al*.* 2014).

Such dynamic relationships between social networks and parasite acquisition suggest that broader socioecological contexts may determine the circumstances under which social life may be beneficial versus detrimental to infectious disease risk. The variant nature of the human-macaque interface may present such contexts and thereby influence parasite acquisition by altering the structure and connectedness of macaque social networks. For instance, higher frequencies of interactions with humans and/or changes to macaque movement or foraging behavior in landscapes altered by anthropogenic disturbance, by constraining the time available for macaques to engage in social interactions (Dunbar 1992; Kaburu et al*.* 2019; Marty et al. 2019), may lead to more modular, substructured social networks which may present bottlenecks to parasite transmission. On the other hand, such interactions or anthropogenic changes may present environmental stressors to the macaques (e.g., Barbary macaques: Carne et al*.* 2017; Marechal et al*.* 2011, 2016), in which individuals possessing strong and diverse social networks may benefit by being socially buffered against infection. In summary, future research may focus on establishing the precise mechanism(s) of super spreading, social bottlenecking, or stress-induced acquisition of parasites, through which social or contact network connectedness may influence parasite transmission dynamics at human-macaque interfaces.

### Bipartite and Multimodal Networks

Social networks have proven exceptionally useful to model the acquisition and transmission of parasites. Yet by themselves, they are somewhat limited in not capturing heterogeneity at higher organizational scales, for instance, across interactions between different components of a social or ecological system. This may be the reason why most epidemiological studies implementing network approaches to model heterogeneity in contact patterns have focused on either human systems or more recently wildlife systems (reviewed above), but almost never at the human-wildlife interface. Bipartite and multimodal networks, which establish connections between different interlinked components of a system, may prove especially useful in this regard (Dormann et al*.* 2017; Kane and Alavi 2008; Latapy et al*.* 2008; Finn et al*.* 2019). Recently, bipartite networks are beginning to feature in ecological and evolutionary research (reviewed in Bascompte and Jordano 2014; Cagnolo et al*.* 2011; Dormann et al*.* 2017), as illustrated by their being used to model marine food webs (Rezende et al*.* 2009), mutualistic interactions between flowers and seed-dispersing animal pollinators (Spiesman and Gratton 2016; Stang et al*.* 2009; Vazquez et al*.* 2009), and, more pertinently, host-parasitoid relationships (Laliberte and Tylianakis 2010; Poulin et al*.* 2013). For networks that combine links both within and across system components, some researchers have coined the term “multimodal networks,” aka “multilayer” or “multislice networks” (Kane and Alavi 2008; Finn et al*.* 2019).

Their potential to model the connections of complex systems make bipartite and multimodal networks highly relevant tools for infectious disease research at human-wildlife interfaces. Yet to our knowledge, they have not been extensively used in this context. In the context of primates and EIDs, Gomez et al. (2013) used a combination of bipartite network construction and social network analytical tools to identify primate species that are likely to harbor EIDs. They constructed a bipartite network that linked each primate species with each parasite isolated from them. From this, they projected a unipartite “social” network in which primate species as nodes were linked to each other by edges weighted by the number of parasites they shared. They then revealed that species that were highly central in this “primate-parasite” network were also the most likely to function as “super spreaders” of EIDs to humans or at least most likely to share those EIDs with humans, suggesting potential conservation implications as well. However, only one macaque species—the toque macaque—was among the top 10 most central primates in this network. So, the extent to which macaques pose threats as transmitters of EIDs remains unclear [and more generally reflective of a knowledge gap in infectious disease research in East and Southeast Asia (Hopkins and Nunn 2007)], though this is undoubtedly likely to vary across contexts.

As reviewed in the previous section, humans and macaques engage in a variety of interactions at interfaces, some forms of which have already been linked to the acquisition and transmission of parasites (summarized in Table 13.1). To assess the mechanistic bases of such transmission, we recommend that future studies, implementing a CNHS approach, focus on constructing bipartite and multimodal networks based on intra- and interspecies interactions and spatial overlap at human-macaque interfaces. For such networks, the choice of system components may also be informed by the characteristics of the parasite studied (Craft 2015). For instance, the transmission of RNA respiratory and retroviruses between humans and macaques may require intense contact events such as bites and scratches and the exchange of body fluids like blood and saliva. Yet their fast mutation rates and short generation times may make them difficult to detect. So, multimodal networks of mild and severe contact aggressive interactions among and between humans and macaques over shorter durations of time may be useful for these purposes. On the other hand, the transmission of gastrointestinal helminths, protozoa, and enteric bacteria may require just subtle interactions such as human provisioning of macaques, acquisition from soil and anthropogenic surfaces, and macaque social grooming and contact huddling interactions, any or all of which would involve fecal-oral transmission (Balasubramaniam et al*.* 2016; Beisner et al*.* 2014, 2016; MacIntosh et al*.* 2012). Given that enteric bacteria may also survive longer in anthropogenic environments such as human-contaminated food, water sources and substrates (Sinton et al*.* 2007), moist soil (Kilonzo et al*.* 2011), and livestock (Craft 2015; Rwego et al*.* 2007; VanderWaal et al*.* 2014), unraveling their transmission routes may involve the construction of “multipartite” networks connecting contact patterns and spatial overlap between these biotic and abiotic factors. An even higher level of complexity may be required to detect the transmission routes of vector-borne malarial parasites like *Plasmodium knowlesi* (Abkallo et al*.* 2014; Huffman et al*.* 2013b; Lee et al*.* 2011). These may depend heavily on geospatial variation in the distribution and overlap of humans, reservoir macaques, and other host wildlife populations, anopheline vectors, and a host of environmental factors that may be conducive to the completion of both vector and pathogen life histories (Lee et al*.* 2011; Moyes et al*.* 2014).

### Microbial Transmission Networks

More recently, the phylogenetic relationships of symbiotic gut microbes isolated from animal hosts have been used to construct microbial transmission networks (VanderWaal and Ezenwa 2016). Such networks offer special advantages to detecting the potential transmission pathways of parasites that spread through the fecal-oral route (Sears et al*.* 1950, 1956; Tenaillon et al*.* 2010). Symbiotic microbes like gastrointestinal *E. coli* are present in almost every individual, have shared evolutionary histories with intestinal pathogens, and are typically acquired via fecal-oral routes (Caugant et al*.* 1981; Sears et al*.* 1950, 1956). So, if two individuals have genotypically similar or identical strains of *E. coli*, they are likely to have either shared the strain via fecal-oral transmission, which may occur either through direct social contact or through using a common environmental source (Chiyo et al*.* 2014; Springer et al*.* 2016; VanderWaal et al*.* 2013, 2014). Further, they rarely (if ever) alter the behavior of the host (VanderWaal et al*.* 2014), which allows researchers to potentially detect subtle transmission events that may signal the potential for a more devastating outbreak. Limited research to date has revealed strong links between the degree of dyadic similarity in *E. coli* and the frequency of animal space use overlap and/or social contact patterns (Balasubramaniam et al*.* 2018; Chiyo et al*.* 2014; Springer et al*.* 2016; VanderWaal et al*.* 2013, 2014). Recently, a study on captive rhesus macaques established that macaques in the same social network communities were more likely to share strains of *E. coli* among themselves than they were to macaques from other communities (Balasubramaniam et al*.* 2018). Previous assessments at human-primate interfaces have revealed that the population genetic structure of gut *E. coli* from great ape populations living in human-perturbed habitats was more similar to *E. coli* from humans and livestock than they were to bacteria. Such findings encourage future efforts to construct microbial transmission networks between humans and overlapping macaque populations, which may serve as models to gauge the potential transmission pathways of parasites with greater precision. However, the lack of discernible mortality or sickness behaviors associated with acquiring non-pathogenic microbes makes them models, rather than accurate forecasters, for parasite transmission.

### Agent-Based Modeling

When data on biological systems are either unavailable or incomplete, mathematical models offer ways of dealing with such inadequacies. In simple terms, a model may be thought of as a simplified version of a study system used to better understand it (Epstein and Axtell 1996; Minsky 1965). Thus, the complexity of a model has to be lower than that of the study system; otherwise its usefulness is lost. The main advantage of a computational model is that it can be tested infinitely by recreating or simulating a situation in the same way many (thousands of) times by adding, removing, or varying parameters and measuring emergent characteristics. These results are then usually compared with findings from empirical data to confirm or reject the tested hypotheses and accept (at least temporarily) the one for which the simulations best explain the data (Epstein and Axtell 1996; Minsky 1965). Furthermore, outcomes from these models may reveal complex or unexpected effects which may be different from, or even go undetected, based on a priori predictions. This might in turn provide a stronger basis to make other predictions, including those related to the relative role(s) of different predictive factors, in future assessments of biological systems (Epstein and Axtell 1996; Minsky 1965).

Unsurprisingly, the bottom-up approaches of simulated models have made them exceptionally useful tools to understand the acquisition and transmission of parasites. The first mathematical models of parasite transmission did not implement social network approaches: virtual individuals moved and interacted randomly in their environments (Wilensky and Stroup 1999). From individual characteristics (e.g., age, sex, hierarchical rank) and basic interaction rules (social contact, spatial proximity, conflicts, etc.), these tried to assess how the more global phenomena of parasite prevalence and outbreak potential emerge in a system (Romano et al*.* 2016; Rushmore et al. 2014). In these models, only the R_0_, the initial number of infected agents and their interaction rates, mattered. R_0_ is the basic reproduction number used to quantify the transmission potential of a parasite, defined as the number of secondary infections caused by a single infected individual introduced into a population made up entirely of susceptible individuals. However, following the acknowledgment that individuals do not move or interact randomly, social networks have been integrated into these models during the last decade or so (Griffin and Nunn 2012; Huang and Li 2007; McCabe and Nunn 2018; Nunn 2009).

Expanding beyond homogenous populations that contain only susceptible individuals that interact randomly, recent studies have integrated network approaches with a classic set of individual-based models used in epidemiology that classify individuals or “agents” into moving between susceptible infected and resistant (or SIR) classes or compartments (Bansal et al. 2007; Brauer 2008; Grimm and Railsback 2005; Kohler and Gumerman 2000). Such integrated network-based SIR models now form the basis of many epidemiological assessments in primate systems (Griffin and Nunn 2012; Kohler and Gumerman 2000; McCabe and Nunn, 2018; Rushmore et al*.* 2014). For instance, the likelihood of parasite transmission from A to B may be a function of (1) whether A is already infected, (2) the likelihood of a link between A and B in their social network, and (3) the per-contact transmission probability “Beta.” Simulations are run until set criteria are reached, e.g., either the extinction or saturation of infection throughout the group, at which time the average outbreak size or *R*_infinity_ is calculated (Diekmann et al*.* 1998; Keeling 1999). The implemented social network might be theoretical (Sueur et al. 2012), based on empirical data (Romano et al*.* 2016; Rushmore et al. 2014), or both (Griffin and Nunn 2012) depending on the aims of the study.

In a comparative evolutionary analysis that used both natural primate datasets and simulated networks, Griffin and Nunn (2012) used an SIR model to reveal how increased community modularity in the social network, despite a larger group size, negatively impacts parasite success. In a more applied example of the implementation of agent-based models, Rushmore et al. (2014) modeled the social network of a wild population of chimpanzees in order to target specific individuals to vaccinate. Based on their social centrality, they revealed that it was sufficient to target fewer, more central individuals for the same result regarding controlling the spread of infection. In this study, social network position was a model parameter, but it might also be the study object if the model includes feedback loops between the social network and parasite acquisition. Corner et al*.* (2003), for instance, not only showed that social networks of Australian possums (*Trichosurus vulpecula*) have an effect on the transmission rate of tuberculosis (TB) but also that the spread of TB had a feedback effect on the social network: infected possums showed higher proximity degree and betweenness centrality than non-infected possums. To our knowledge, agent-based models have not been applied to assess parasite transmission at human-macaque interfaces. In order to do so, we recommend that future research implement the SIR approach, but focus on using multimodal network models in the place of social networks which may account for an added level of complexity to model the heterogeneity between human-macaque and macaque-macaque interactions.

## Conclusions

Our goals in writing this chapter were threefold. First, we wanted to convey how, despite a recent surge in primate infectious disease ecological research during the last two decades, relatively few efforts have focused on the impact of humans and anthropogenic factors on disease risk in wild primates. Second, we hope to have illustrated how our current knowledge of human-macaque interactions in particular present “starting points” from which long-term, in-depth assessments of the ecology of parasite acquisition and transmission at human-primate interfaces may be conducted. We believe that such efforts would need to integrate ethnoprimatology and infectious disease ecology in order to be successful. Finally, we hope to have convinced readers of how research on infectious disease ecology at human-macaque interfaces are also in need of the implementation of both novel conceptual frameworks (e.g., One Health, Coupled Systems) and cutting-edge analytical approaches (e.g., Network Analyses, mathematical modeling). Such approaches not only bolster the scope of epidemiological research but are also imperative for the conservation and management of both threatened and potentially problematic free-living primate (and indeed other wildlife) populations.
